# PHYSICAL ACTIVITY AND IMPACT ON PSYCHOLOGICAL AND BEHAVIOR SYMPTOMS ASSOCIATED WITH DEMENTIA

**DOI:** 10.1093/geroni/igad104.0701

**Published:** 2023-12-21

**Authors:** Brittany Drazich

**Affiliations:** University of Maryland Baltimore, Baltimore, Maryland, United States

## Abstract

The aim of this study was to examine the impact of physical activity during hospitalization on behavioral and psychological symptoms of dementia among older adults, after controlling for covariates (age, gender, race, comorbidities, care interactions, and treatment group) and examine the association of physical and behavioral and psychological symptoms of dementia among hospitalized older adults living with dementia. Data from the first 365 participants in the FFC-AC-EIT study was used. Physical activity was measured over 24 hours and was based on the Physical Activity Survey which included the following activities: walking, wheeling, bathing, dressing, feeding, grooming, and toileting. Behavioral and psychological symptoms associated with dementia were based on the Neuropsychiatric Inventory (NPI). The mean age of participants was 83.18 years, the majority was female (63.6%) and White (69.4%), the mean NPI was 1.28(SD=1.85) and mean activities was 8(SD=5). A total of 16% of individuals performed no activity. The most common activity was eating and the least commonly performed was self-propelling in a wheelchair. A total of 40% engaged in walking and 60% performed some bathing and dressing while hospitalized. After controlling for covariates, physical activity explained an additional 2% of the variance in NPI scores (beta=-.13., p=.05). There was no evidence that engaging patients in physical activity resulted in behavioral symptoms and activity may help to decrease these symptoms. Continued focus is needed to engage older adults living with dementia when hospitalized to engage in routine physical activity by performing activities of daily living.

